# The effects of dietary supplementation with mushroom or selenium enriched mushroom powders on the growth performance and intestinal health of post-weaned pigs

**DOI:** 10.1186/s40104-022-00808-x

**Published:** 2023-01-11

**Authors:** Alison Dowley, Torres Sweeney, Eadaoin Conway, Stafford Vigors, Marion T. Ryan, Supriya Yadav, Jude Wilson, John V. O’Doherty

**Affiliations:** 1 School of Agriculture and Food Science, University College Dublin, Belfield, Dublin 4, Ireland; 2 School of Veterinary Medicine, University College Dublin, Belfield, Dublin 4, Ireland; 3 Mbio, Monaghan Mushroom Group, Tyholland, Co.Monaghan Ireland

**Keywords:** *Agaricus bisporus*, Gastrointestinal microbiota, Mushroom powder, Organic selenium, Pig, Zinc oxide

## Abstract

**Background:**

There is an urgent need to identify natural bioactive compounds that can enhance gastrointestinal health and promote pig growth performance in the absence of pharmacological levels of zinc oxide (ZnO). The objectives of this study were to: 1) compare the effects of mushroom powder supplemented with inorganic selenium (inSeMP) to mushroom powder enriched with organic selenium (orgSeMP) to pharmacological levels of ZnO on growth performance and faecal scores (FS) for the first 21 d post-weaning (Period 1); and 2) compare the molecular and microbial effects of inSeMP and orgSeMP in these pigs on d 39 post-weaning (Period 2).

**Methods:**

In Period 1, pigs (3 pigs/pen; 8 pens/treatment) were assigned to: (1) basal diet (control); (2) basal diet + zinc oxide (ZnO) (3100 mg/kg d 1–14, 1550 mg/kg d 15–21); (3) basal diet + mushroom powder supplemented with inorganic selenium (inSeMP) containing selenium (selenite) content of 0.3 mg/kg feed; (4) basal diet + mushroom powder enriched with organic selenium (orgSeMP) containing selenium (selenocysteine) content of 0.3 mg/kg feed. Mushroom powders were included at 6.5 g/kg of feed.

**Results:**

In Period 1, there was no effect of diets on average daily gain (ADG) and gain:feed (G:F) ratio (*P* > 0.05). The orgSeMP supplemented pigs had a lower average daily feed intake (ADFI) compared to all other groups (*P* < 0.05). The ZnO supplemented pigs had reduced FS compared to the basal and mushroom group, while the orgSeMP supplemented pigs had lower FS compared to the basal group during the 21 d experimental period (*P* < 0.05). In Period 2, there was no effect of diets on ADFI, ADG and G:F ratio (*P* > 0.05). The orgSeMP supplementation increased the caecal abundance of bacterial members of the Firmicutes and Bacteroidetes phylum, including *Lactobacillus, Agathobacter, Roseburia,* and *Prevotella* and decreased the abundance of *Sporobacter* compared to the basal group, while inSeMP increased the caecal abundance of *Prevotella* and decreased the caecal abundance of *Sporobacter* compared to the basal group (*P* < 0.05). Dietary supplementation with inSeMP increased expression of *TLR4* and anti-inflammatory cytokine gene *IL10* and decreased nutrient transporter gene *FABP2* compared to the orgSeMP group (*P* < 0.05).

**Conclusion:**

OrgSeMP is a novel and sustainable way to incorporate selenium and β-glucans into the diet of weaned pigs whilst improving FS and modulating the caecal microbiota.

**Supplementary Information:**

The online version contains supplementary material available at 10.1186/s40104-022-00808-x.

## Background

In commercial pig production systems, weaning involves complex dietary, social, and environmental stressors which cause a transient reduction in feed intake [1]. This contributes to adverse gut morphological and functional changes which leads to epithelial permeability and upregulation of proinflammatory cytokines [2]. As a result, the digestive and absorptive capacity of the small intestine is impaired, with consequent lower nutrient absorption and reduced energy availability [3]. Intestinal inflammation, mediated by the upregulation of proinflammatory cytokines, promotes the intestinal proliferation of pathogenic bacteria like *Escherichia coli* leading to gut dysbiosis and post-weaning diarrhoea (PWD) [4].

Dietary supplementation with zinc oxide (ZnO) at pharmacological levels (2000 to 3100 mg/kg) during the immediate post-weaning period is an industry-wide practice to alleviate the negative impact of weaning on pig performance and gastrointestinal functionality and health [4, 5]. However, from June 2022, pharmacological doses of ZnO will no longer be authorized in the European Union [6, 7]. Therefore, there is increasing pressure in identifying natural bioactive compounds that may support growth, enhance beneficial microbial populations, and prevent diarrhoea similar to that of ZnO.

Selenium is an essential trace nutrient and has an integral role in promoting immune function, growth performance and meat quality [8, 9]. Selenium has also demonstrated bacterial-modulating activities, including increased *Lactobacilli* spp. and decreased *E. coli* spp. counts in the caecum of broilers [10] and faeces of pigs [11]. Dietary supplementation with selenium enriched yeast for 21 d post-weaning improved pig growth performance and reduced the production of cytokines associated with inflammation, including TNF-α and IL-6, in the liver and thymus of pigs exposed to oxidative stress [12].

Selenium occurs in both inorganic and organic forms [13]. Inorganic selenium is mainly used in the form of sodium selenite and is the most widely used selenium supplement in animal diets. However, replacing inorganic selenium in animal diets with an organic form has received considerable interest in recent years as organic sources of selenium exhibit lower toxicity and higher bioavailability in animals compared to inorganic sources [14]. While the National Research Council recommends 0.15–0.30 mg/kg of added selenium in weaned pig diets, the total maximum level of dietary selenium in swine diets is 0.5 mg/kg [15, 16]. Clinical selenium deficiency is a rare occurrence in commercial pig production, however sub-clinical selenium deficiency in young animals is more common and may be responsible for decreased pig health and performance [17]. As the inclusion levels of selenium in pig diets are bound by legal standards, incorporating selenium sources with high bioavailability, such as organic selenium, into pig diets may be an effective method of increasing selenium uptake in pigs.

The utilization of mushrooms as novel feed additives in animal diets is gaining considerable interest in recent years. Mushrooms are a rich natural source of bioactive compounds, such as phenolics, lectins, terpenoids, ergosterols and β-glucans [18, 19]. β-glucans have well recognized anti-inflammatory, antioxidant and immunomodulatory properties [20–22]. β-glucans are non-digestible polysaccharides and therefore have the potential to modulate the gastrointestinal microbiota of pigs [23]. Yeast β-glucans in the diet of weaned pigs can increase faecal *Lactobacillus* populations [24] and decrease faecal *Escherichia coli* numbers [25] while also improving growth performance [26]. Mushrooms also offer a unique opportunity to incorporate organic selenium into the diet of pigs. Mushrooms are irrigated with sodium selenite solution as a method of selenium enrichment [27]. Sodium selenite, the inorganic form of selenium, is taken up by mushrooms through phosphate transporters and reduced to selenide, before being converted to selenocysteine, the organic form of selenium [28]. Thus, the first objective of this study was to compare the effects of mushroom powder supplemented with inorganic selenium (inSeMP), mushroom powder enriched with organic selenium (orgSeMP) and pharmacological levels of ZnO on growth performance of pigs during the first 21 d post-weaning (Period 1). The second objective of this study was to compare the molecular and microbial effects of inSeMP and orgSeMP supplementation in these pigs on d 39 post-weaning (Period 2). It was hypothesised that orgSeMP would be more effective than inSeMP at enhancing growth performance and gastrointestinal health of post-weaned pigs.

## Materials and methods

### Experimental design and diets

At weaning (28 d), 96 pigs (48 male and 48 female), progeny of Meatline boars × (Large White × Landrace sows) with an average weight of 6.8 kg ± 0.86 (standard deviation (SD)) were selected from a commercial pig farm. The experiment was designed as a complete randomised block and was split into two periods. Period 1 investigated the effects of inSeMP and orgSeMP supplementation on pig growth performance and faecal scores post-weaning (d 0–21) and Period 2 investigated the molecular and microbial effects of inSeMP and orgSeMP supplementation in pig diets on d 39 post-weaning. The pigs were blocked by weaning weight, sex and litter of origin. In Period 1, pigs were assigned to one of four dietary groups. The diets were as follows: (1) basal diet (control); (2) basal diet + ZnO (3100 mg/kg d 1–14, 1550 mg/kg d 15–21); (3) basal diet + inSeMP containing selenium content of 0.3 mg/kg feed; (4) basal diet + orgSeMP containing selenium content of 0.3 mg/kg feed. All other diets contained a selenium (selenite) content of 0.3 mg/kg feed. The inorganic selenium was in the form of sodium selenite and the organic selenium was in the form of selenocysteine. The detection and quantification of amino acids in selenium enriched *Agaricus bisporus* mushrooms was performed using the method of Maseko et al. [27]. The predominant amino acid present was found to be selenocysteine in dried mushroom powder, with modest amounts of selenomethionine identified also. The selenium mushroom powder contained a total selenium concentration of 45.8 mg/kg mushroom powder and was included at 6.5 g/kg to achieve the selenium content of 0.3 mg/kg feed [29] and as a result the β-glucan content of the mushroom diets was 650 mg/kg. Mushrooms were irrigated with sodium selenite solution at intermittent cycles as a method of selenium enrichment [27]. The mushroom powders (*Agaricus bisporus*) were sourced from Monaghan Mushrooms (Tyholland, Co Monaghan, Ireland) and were included at 6.5 g/kg feed. The ZnO was sourced from Cargill (Naas, Kildare, Ireland) and was included at 3100 mg ZnO/kg feed and contained 80% zinc, resulting in an inclusion level of 2500 mg Zn/kg feed. After 15 d the inclusion level of ZnO was halved to 1550 mg ZnO/kg feed. At 21 d post-weaning, the ZnO group was removed from the experiment and seventy-two pigs with an average weight of 12.6 kg ± 2.49 (SD) from (T1), (T3) and (T4) in Period 1 proceeded to Period 2 and were kept on their original diets. The mushroom powder contained 305 g/kg of crude protein, 34 g/kg of ether extract, 2.34 mg/kg selenium and 100 mg/g of β-glucan. The diets were formulated to have equivalent nett energy (10.6 MJ/kg), crude protein (208 g/kg) and standardised ileal digestible lysine (13.0 g/kg). All amino acid requirements were calculated relative to lysine [29]. The composition of diets are presented in Table 1.

**Table 1:** Ingredient and chemical composition of diets

Item	Dietary treatments^1^			
**Ingredients, g/kg unless otherwise stated**	Basal	ZnO	inSeMP	orgSeMP
Sodium selenite, mg/kg	6.5	6.5	6.5	0
Mushroom powder	0	0	6.5	0
Se Mushroom powder	0	0	0	6.5
Ground wheat	357.9	354.8	351.4	351.4
Full-fat soya bean	170	170	170	170
Soya bean meal (48% CP)	105	105	105	105
Whey powder (900 g CP/kg)	50	50	50	50
Zinc oxide	0	3.1	0	0
Soya oil	30	30	30	30
Soybean concentrate	65	65	65	65
Flaked wheat	127.5	127.5	127.5	127.5
Flaked maize	70	70	70	70
*L*-Lysine-HCl	4	4	4	4
*DL*-Methionine	2	2	2	2
*L*-Threonine	1.8	1.8	1.8	1.8
*L*-Tryptophan	0.3	0.3	0.3	0.3
Sodium bicarbonate	2	2	2	2
Monocalcium phosphate	4	4	4	4
Vitamin and mineral premix^2^	2.5	2.5	2.5	2.5
Calcium carbonate (limestone)	6	6	6	6
Salt	2	2	2	2
**Analysed chemical analysis**				
Gross energy, MJ/kg	16.9	16.9	16.8	16.9
Dry matter	899.0	899.5	897.5	898.1
Crude protein	208.0	208.3	208.5	208.5
Lysine, %^3^	1.4	1.4	1.4	1.4
Threonine, %^3^	0.9	0.9	0.9	0.9
Methionine and cysteine, %^3^	0.8	0.8	0.8	0.8
Tryptophan, %^3^	0.3	0.3	0.3	0.3
Standardised ileal digestible lysine^3^	13.0	13.0	13.0	13.0
Crude fat	79.8	80.3	80.2	80.0
Crude fibre	28.0	28.0	28.2	28.3
Neutral detergent fibre	98.9	98.8	99.5	99.3
Ash	46.2	46.1	46.0	46.2
Selenium, mg/kg	0.33	0.29	0.31	0.32
β-glucan, mg/kg	0	0	649.2	649.7

*InSeMP* mushroom powder supplemented with inorganic selenium, *orgSeMP* mushroom powder enriched with organic selenium

^1^Dietary treatments: (1) Basal diet; (2) basal diet + ZnO (3100 mg/kg d 1–14, 1550 mg/kg d 15–21); (3) basal diet + inSeMP containing an inorganic selenium (selenite) content of 0.3 mg/kg feed and a β-glucan content of 650 mg/kg feed; (4) basal diet + orgSeMP containing an organic selenium (selenocysteine) content of 0.3 mg/kg feed and a β-glucan content of 650 mg/kg feed

^2^Provided (per kg diet): 1.8 mg retinol; 0.025 mg cholecalciferol; 67 mg tocopherol; 4 mg menaquinone; 0.1 mg cyanocobalamin; 2 mg riboflavin; 12 mg nicotinic acid; 10 mg pantothenic acid; 250 mg choline chloride; 2 mg thiamine; 0.015 mg pyridoxine; 25 mg copper (copper sulfate); 140 mg iron; 47 mg Manganese; 120 mg Zinc; 0.6 mg iodine; 0.3 mg sulfur

^3^Calculated for the tabulated nutritional composition [30]

### Housing and animal management

The pigs were penned in groups of three according to weight and sex and housed on fully slatted floors (1.68 m × 1.2 m). There were 8 replicate pens used per treatment with 3 pigs in each replicate pen. For the first 7 d, the temperature within the weaner house was controlled at 30 ºC and then reduced by 2 ºC per wk until the temperature reached 26 ºC. The relative humidity was maintained at 65%. The experimental diets were given in mash meal form from four-space feeders and pigs had ad libitum access to these diets immediately after weaning up to the final weighing. The diets were formulated to meet all nutrient requirements recommended by National Research Council [29]. Drinking water was available ad libitum from a drinking nipple. Pigs received no medication throughout the experiment. Body weight (BW) was measured using a portable electronic scale (Prattley, Temuka, New Zealand) on d 1, 21 and 39 and average daily gain (ADG), average daily feed intake (ADFI) and gain to feed (G:F) were calculated on a per-pen basis. The daily feed intake was measured at a pen level by weighing the feed delivered to each feeder. Faecal scores (FS) were assessed twice daily for each individual pen throughout the experimental period to indicate the presence and severity of diarrhoea. The following scoring system was used to assign FS: 1 = hard, 2 = slightly soft, 3 = soft, partially formed, 4 = loose, semi-liquid, 5 = watery, mucous like [31].

### Feed analysis

All the feed samples were milled through a 1-mm screen (Christy and Norris Hammer Mill, Chelmsford, England) and kept for chemical analysis. The gross energy (GE) content was determined using an adiabatic bomb calorimeter (Parr Instruments, Moline, IL, USA) as previously described [32]. The feed was dried for 72 h at 55 °C to determine the dry matter (DM) content of the feed. Feed samples were analysed for crude ash (AOAC.942.05), nitrogen (AOAC.990.03), crude fibre (AOAC.978.10) and crude fat (AOAC.920.39) according to the Association of Official Agricultural Chemists standard procedures [33], and neutral detergent fiber (NDF) was determined according to the method of Van Soest et al. [34]. The MP was analysed for nitrogen (AOAC.990.03), crude fat (AOAC.920.39), selenium and β-glucans. The total glucans of the MP were determined using the kit K-YBGL, purchased from Megazyme (Bray, Co Wicklow, Ireland), following the manufacturer’s recommendations, and as previously described [35]. The total selenium content was measured by Eurofins Food Testing UK Ltd (ICPMS 7800; Wolverhampton, United Kingdom) using the selenium in food method. Briefly, samples were digested by acid solutions (Nitric/Hydrochloric mix) in sealed tubes using heat and pressure in a microwave accelerated reaction system. All samples were measured in duplicate.

### Sample collection

On d 39 of the experiment, 8 pigs per treatment (one pig/pen) received a lethal injection with pentobarbitone sodium (Euthatal Solution, 200 mg/mL; Merial Animal Health, Essex, UK) at a rate of 0.71 mL/kg body weight to the cranial vena cava to humanely euthanise the animals. Sections from the duodenum (10 cm from the stomach), jejunum (60 cm from the stomach) and ileum (15 cm from the caecum) were processed for gut morphological analysis as previously described [36]. Digesta from the caecum was collected and stored in sterile containers (Sarstedt, Wexford, Ireland). This was then snap frozen on dry ice and stored at −80 °C for subsequent 16 S rRNA sequencing and volatile fatty acid (VFA) analysis. In addition, tissue samples were taken from the duodenum, jejunum, and ileum to measure the expression of cytokines, nutrient transporters, mucins, tight junctions, and appetite regulators using quantitative real-time PCR (qPCR). Tissue sections (1 cm) from the duodenum, jejunum, and ileum were cut out, dissected along the mesentery, emptied, and rinsed using sterile phosphate buffered saline (Oxoid, Hampshire, UK). The tissue sections were stripped of the overlying smooth muscle before storage in RNAlater® (5 mL) solution (Applied Biosystems, Foster City, CA, USA) overnight at 4 °C. The RNAlater® was removed before storing the samples at −80 °C.

### Volatile fatty acid analysis

Gas liquid chromatography was used to determine the VFA and branched-chain VFA (BCVFA; isobutyrate, isovalerate and valerate) concentrations in the caecal digesta as described previously by Clarke, Sweeney [37]. 1 g of digesta was diluted with water (2.5 × sample weight) and centrifuged (1400 × *g* for 10 min) using a Sorvall GLC-2B centrifuge (DuPont, Wilmington, DE, USA). 1 mL of supernatant and 1 mL of internal standard (0.05% 3-methyl-n-valeric acid in 0.15 mol/L oxalic acid dihydrate) were mixed with 3 mL of distilled water and then centrifuged for 10 min at (500 × *g*). The supernatant was then filtered through a syringe filter (0.45 polytetrafluoroethylene (TFE)) into a chromatographic sample vial. Approximately 1 µL of this mixture was injected into a Varian 3800 GC (Ontario, Canada) with an ECTM 1000 Grace column (15 m × 0.53 mm I.D) with a film thickness of 1.20 µm. The temperature program was set to the range 75—95 °C which increased by 3 °C/min and 95—200 °C which increased by 20 °C/min, and this was held for 0.50 min. The detector temperature was 280 °C and the injector temperature was 240 °C. The total analysis time was 12.42 min.

### Microbial analyses

#### Microbial DNA extraction

A QIAamp PowerFecal Pro DNA Kit (Qiagen, West Sussex, United Kingdom) was used to extract microbial genomic DNA in accordance with the manufacturer’s instructions. A Nanodrop ND-1000 Spectrophotometer (Thermo Scientific, Wilmington, DE, USA) was used to measure the quantity and quality of the DNA.

#### Illumina sequencing

Bacterial DNA was extracted from the caecal digesta samples and high-throughput sequencing of the V3—V5 hypervariable region of the bacterial 16S rRNA gene was performed on an Illumina MiSeq platform according to their standard protocols (Eurofins Genomics, Ebersberg, Germany).

#### Bioinformatic

The bioinformatic assessment of the sequences were conducted by Eurofins Genomics (Ebersberg, Germany) using the package (version 1.9.1) Quantitative Insights into Microbial Ecology [38]. All the raw reads passing the standard Illumina chastity filter were demultiplexed in accordance with their index sequences (read quality score > 30). The primer sequences were clipped from the beginning of the raw forward/reverse reads. If primer sequences did not match perfectly, read pairs were eliminated to retain only high-quality reads. Paired-end reads were then merged, to get a single, longer read that covers the complete target region using the software FLASH 2.2.00 [39]. The pairs were merged with the lowest overlap size of 10 bp to decrease false-positive merges. The forward read was only kept for the subsequent assessment steps when merging was not viable. Merged reads were quality filtered in accordance with the expected and known length variations of the V3—V5 region (ca. 445 bp). The ends of retained forward reads were clipped to a complete read length of 285 bp to eliminate low quality bases. Merged and retained reads comprising of ambiguous bases were removed. The filtered reads were then used for profiling of the microbiome. Chimeric reads were detected and deleted based on the de-novo algorithm of UCHIME [40] as implemented in the VSEARCH package [41]. The remaining set of high-quality reads were then processed using minimum entropy decomposition (MED) to partition reads to operational taxonomic units (OTU) [42, 43]. DC-MEGABLAST alignments of cluster representative sequences to the NCBI nucleotide sequence database were carried out for the taxonomic assignment of every OTU. A sequence identity of 70% across a minimum of 80% of the representative sequence was the minimal prerequisite for considering reference sequences. Abundances of bacterial taxonomic units were normalized using lineage-specific copy numbers of the appropriate marker genes to enhance estimates [44].

The data matrix was made up of the normalized OTU table in combination with the phenotype metadata and phylogenetic tree. The data matrix was then loaded into the phyloseq package in R (http://www.r-project.org; version 3.5.0). Differential abundance analysis was carried out on tables extracted from the phyloseq object at phylum, family, genus and species level. The model assessed the effect of ‘group’, with the individual pig being the experimental unit. Eight pigs per group were used for the statistical analysis of the relative bacterial abundances.

### Gene expression in the small intestine

#### RNA extraction and cDNA synthesis

Total RNA was extracted from duodenal and ileal tissues using TRI Reagent (Sigma-Aldrich, St. Louis, MO, USA) in accordance with the manufacturer’s guidelines as previously described [45]. The total RNA (2 μg) was reverse transcribed using a High-Capacity cDNA Reverse Transcription Kit (Applied Biosystems, Foster City, CA, USA) and oligo (dT) primers in a final reaction volume of 40 μL, in accordance with manufacturer’s guidelines. The cDNA was then made up to a volume of 360 μL with nuclease-free water.

#### Quantitative real-time polymerase chain reaction (qPCR)

The qPCR reaction mixture (20 μL) consisted of GoTaq qPCR Master Mix (10 μL) (Promega, Madison, WI, USA), forward and reverse primers (5 μmol/L, 1.2 μL), nuclease-free water (3.8 μL) and cDNA (5 μL). All the qPCR reactions were carried out in duplicate on the 7500 ABI Prism Sequence detection System (Applied Biosystems, Foster City, CA, USA). The cycling conditions consisted of a denaturation step of 95 °C for 10 min which was followed by 40 cycles of 95 °C for 15 s and then 60 °C for 1 min. All the primers were designed using the Primer Express Software (Applied Biosystems, Foster City, CA, USA) and made by MWG Biotech UK Ltd (Milton Keynes, UK) and are all described in Table 2. Dissociation curves were created to verify the specificity of the subsequent PCR products. The qPCR assay efficiencies were determined by plotting the cycling threshold (CT) values resulting from fourfold serial dilutions of cDNA against their arbitrary quantities and only assays demonstrating 90%–110% efficiency and single products were accepted in this analysis. Normalised relative quantities were obtained using the software, qbase PLUS (Biogazelle, Ghent, Belgium) from stable reference genes; H3 histone family member 3A (*H3F3A)* and tyrosine 3-monooxygenase/tryptophan 5-monooxygenase activation protein zeta (*YWHAZ)* (duodenum and jejunum), Actin beta (*ACTB)* and *H3F3A* (ileum). These genes were selected as reference genes based on their M value (< 1.5) generated by the GeNorm algorithm within GeNorm. The genes analyzed in the current study are as follows: protein transporter, solute carrier family 15 member 1 (*SLC15A1;* previously known as *PEPT1*); fatty acid transporter 2 (*FABP2)*; glucose transporters solute carrier family 2 member 2 (*SLC2A2*; previously known as *GLUT2*) and solute carrier family 2 member 5 (*SLC2A5*; previously known as *GLUT5*); appetite regulator Cholecystokinin *(CCK)*, neuropeptide Y (*NPY)*, glucagon-like peptide-1 (*GLP1)* and peptide YY (*PYY)*; cytokines tumor necrosis factor alpha (*TNF*α)*,* Interleukin 6 *(IL6),* Interleukin 10 *(IL10),* Interferon gamma *(IFNG),* Transforming growth factor beta 1 (*TGFB1)* and Interleukin 17 (*IL17)*; C-X-C motif chemokine ligand 8 (*CXCL8)* (previously known as Interleukin 8 (*IL8*); Mucin 2 (*MUC2)* and Mucin 1 (*MUC1)*; tight junctions Claudin 3 (*CLDN3)* and Claudin 1 (*CLDN1)*; Toll like receptor 4 (*TLR4)*; selenoproteins iodothyronine deiodinase 1 (*DIO1)*, selenoprotein P (*SELENOP)* and thioredoxin reductase 1 (*TXNRD1)*.

**Table 2:** Panel of porcine oligonucleotide primers used for real-time PCR

Target gene	Accession No.	Primers (5'→3')	Amplicon length, bp
*IL6*	NM_214399.1	F: GACAAAGCCACCACCCCTAA	69
		R: CTCGTTCTGTGACTGCAGCTTATC	
*CXCL8*	NM_213867.1	F: TGCACTTACTCTTGCCAGAACTG	82
		R: CAAACTGGCTGTTGCCTTCTT	
*IL10*	NM_214041.1	F: GCCTTCGGCCCAGTGAA	71
		R: AGAGACCCGGTCAGCAACAA	
*IL17A*	NM_001005729.1	F: CCCTGTCACTGCTGCTTCTG	57
		R: TCATGATTCCCGCCTTCAC	
*IFNG*	NM_213948.1	F: TCTAACCTAAGAAAGCGGAAGAGAA	81
		R: TTGCAGGCAGGATGACAATTA	
*TNFα*	NM_214022.1	F: TGGCCCCTTGAGCATCA	68
		R: CGGGCTTATCTGAGGTTTGAGA	
*TGFB1*	NM_214015.1	F: AGGGCTACCATGCCAATTTCT	101
		R: CGGGTTGTGCTGGTTGTACA	
*TLR4*	NM_001293317.1	F: TGCATGGAGCTGAATTTCTACAA	140
		R: GATAAATCCAGCACCTGCAGTTC	
*MUC1*	XM_001926883.1	F: ACACCCATGGGCGCTATGT	68
		R: GCCTGCAGAAACCTGCTCAT	
*MUC2*	XM_021082584.1	F: CAACGGCCTCTCCTTCTCTGT	70
		R: GCCACACTGGCCCTTTGT	
*CLND1*	NM_001244539.1	F: CTGGGAGGTGCCCTACTTTG	72
		R: TGGATAGGGCCTTGGTGTTG	
*CLND3*	NM_001160075.1	F: GAGGGCCTGTGGATGAACTG	65
		R: GAGTCGTACACTTTGCACTGCAT	
*CCK*	NM_214237.2	F: GGACCCCAGCCACAGAATAA	61
		R: GCGCCGGCCAAAATC	
*FABP2*	NM_001031780.1	F: CAGCCTCGCAGACGGAACTGAA	102
		R: GTGTTCTGGGCTGTGCTCCAAGA	
*SLC2A2/GLUT2*	NM_001097417.1	F: CCAGGCCCCATCCCCTGGTT	96
		R: GCGGGTCCAGTTGCTGAATGC	
*SLC2A5/GLUT5*	XM_021095282.1	F: CCCAGGAGCCGGTCAAG	60
		R: TCAGCGTCGCCAAAGCA	
*SLC5A1/SGLT1*	NM_001164021	F: GGCTGGACGAAGTATGGTG	153
		R: ACAACCACCCAAATCAGAGC	
*SLC15A1/PEPT1*	NM_214347.1	F: GGATAGCCTGTACCCCAAGCT	73
		R: CATCCTCCACGTGCTTCTTGA	
*NPY*	NM_001256367.1	F: CAGGCAGAGATACGGAAAACG	71
		R: TCCGTGCCTCTCTCATCAAG	
*GLP1*	NM_001256594.1	F: CAGTGCAGAAATGGCGAGAA	61
		R: GGTGGAGCCTCAGTCAGGAA	
*PYY*	XM_005668763.1	F: CTCCTGATTCGGTTTGCAGAA	61
		R: GGACAGGAGCAGCAGGAAGA	
*SELENOP*	NM_001134823.1	F:CAGGCCAGCTGATACCTGTGT	83
		R:TTAGAATATCCTTCTTTCTCCAGTTTTACTC	
*DIO1*	NM_001001627.1	F:GGCTCTGGGTGCTCTTTCAG	69
		R:CAGGAAACAATGTCATGAGCACTT	
*TXNRD1*	NM_214154.3	F:CACCGTGACGGACTCAAAACT	72
		R:GCTTGAGGCTGGTGACTTCAT	
*H3F3A*	NM_001014389.2	F:CATGGCTCGTACAAAGCAGA	136
		R:ACCAGGCCTGTAACGATGAG	
*ACTB*	AY550069.1	F:CAAATGCTTCTAGGCGGACTGT	75
		R:TCTCATTTTCTGCGCAAGTT	
*YWZHAZ*	XM_001927228.1	F:GGACATCGGATACCCAAGGA	71
		R:AAGTTGGAAGGCCGGTTAATTT	

### Gut morphological analysis

Standard paraffin embedding techniques were used to prepare the small intestinal tissue for gut morphological analysis, as previously described [36]. A light microscope with an image analyzer (Image-Pro Plus; Media Cybernetics, Oxon, UK) was used to measure the villus height (VH), crypt depth (CD) and villus height to crypt depth ratio (VH:CD). Fifteen measurements of villi and crypt were taken for each section. The VH was measured from the crypt-villus junction to the tip of the villus, and CD was measured from the crypt-villus junction to the base. Results are expressed as mean VH or CD in μm.

### Statistical analysis

All data on growth performance, gastrointestinal morphology, gene expression and VFA were checked for normality using the univariate procedure of Statistical Analysis Software (SAS) 9.4 and transformed, if required. The general linearized model (GLM) procedure within SAS was used to analyze the data on growth performance, gastrointestinal morphology, gene expression (Bonferroni adjusted *P* < 0.05), and VFA concentrations. The model examined the effects of treatment, using weight at weaning as a covariate. FS were averaged for every 3 d for the first 21 d and analysed using the PROC MIXED procedure of SAS. The model examined the effect of treatment, time and the associated interaction and using weight at weaning as a covariate. The microbiome data were analysed using PROC GLIMMIX. Results are presented using Benjamini–Hochberg (BH) adjusted *P*-values. The pen was the experimental unit for growth performance and FS, while the individual pig was the experimental unit for gastrointestinal morphology, gene expression, microbiome and VFA data. The results are presented as least-square means with their standard errors. The probability level that denotes significance is *P* < 0.05.

## Results

### Pig performance and faecal consistency in Period 1 (d 0–21)

The effects of dietary supplementation on ADG, ADFI and G:F ratio to d 21 post-weaning are presented in Table 3. While the orgSeMP supplemented pigs had a lower ADFI compared to all other groups (*P* < 0.05), there was no difference in ADG, G:F and final BW between groups over the 21-day period (*P* > 0.05).

**Table 3:** Effect of dietary treatment on pig growth performance (d 0–21; least-square mean values ± SEM)

Item	**Dietary treatments**^**1**^				**SEM**	***P*-value**
	**Basal**^**2**^	**ZnO**^**2**^	**inSeMP**^**2**^	**orgSeMP**^**2**^		
Initial weight, kg	6.81	6.80	6.79	6.79	0.175	0.999
Final body weight, kg	12.58	12.55	12.91	12.22	0.326	0.688
ADG, kg/d	0.28	0.26	0.27	0.27	0.014	0.630
ADFI, kg/d	0.39^b^	0.38^b^	0.39^b^	0.35^a^	0.010	0.002
Gain:feed, kg/kg	0.68	0.62	0.65	0.72	0.048	0.488

*ADG* average daily gain, *ADFI* average daily feed intake, *inSeMP* mushroom powder supplemented with inorganic selenium, *orgSeMP* mushroom powder enriched with organic selenium

^1^Dietary treatments: (1) Basal diet; (2) basal diet + ZnO (3100, mg/kg d 1–14, 1550, mg/kg d 15–21); (3) basal diet + inSeMP containing an inorganic selenium (selenite) content of 0.3 mg/kg feed and a β-glucan content of 650 mg/kg feed; (4) basal diet + orgSeMP containing an organic selenium (selenocysteine) content of 0.3 mg/kg feed and a β-glucan content of 650 mg/kg feed

^2^A total of 8 replicates were used per treatment group

^a−b^Mean values within a row with unlike superscript letters were significantly different (*P* < 0.05)

**Fig. 1:**
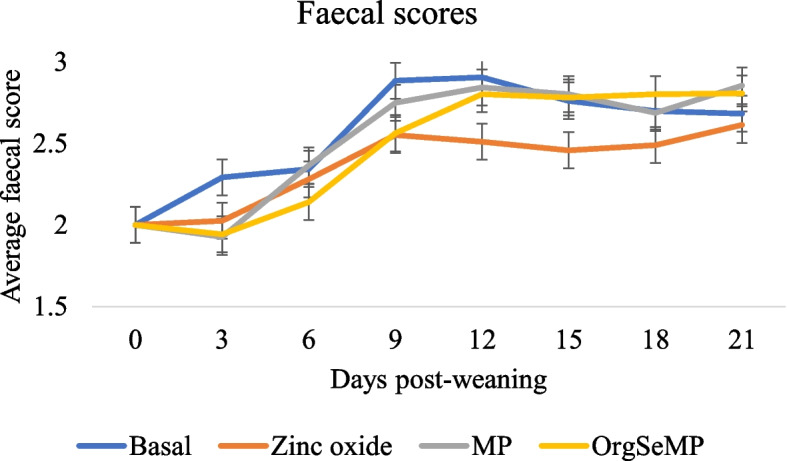
Effect of dietary treatment on faecal scores from day 0 to 21 post-weaning. Values are means, with their standard errors represented by vertical bars. Scale from 1 to 5: 1 = hard, firm faeces; 2 = slightly soft faeces; 3 = soft, partially formed faeces; 4 = loose, semi-liquid faeces and 5 = watery, mucous-like faeces. A total of 8 replicates were used per treatment (replicate = pen, 3 pigs/pen). Treatment (*P* < 0.05), Time (*P* < 0.05), Treatment × Time (*P* > 0.05). Abbreviations: InSeMP, mushroom powder supplemented with inorganic selenium; orgSeMP, mushroom powder enriched with organic selenium

The effects of dietary supplementation on FS from day 0 to 21 post-weaning are presented in Fig. 1. There was no treatment × time interaction on FS (*P* > 0.05). Overall the ZnO supplemented pigs had reduced FS compared to the basal and inSeMP group during the 21-day experimental period (*P* < 0.05). The orgSeMP supplemented pigs had lower FS compared to the basal group (*P* < 0.05).

### Pig performance in Period 2 (d 39)

The effect of dietary supplementation on ADG, ADFI, G:F ratio and final BW is presented in Table 4. Overall, there was no difference in ADG, ADFI, G:F ratio and final BW between groups (*P* > 0.05).

**Table 4:** Effect of dietary treatment on pig growth performance (d 21–39; least-square mean values ± SEM)

Item	**Dietary treatments**^**1**^			**SEM**	***P*-value**
	**Basal**^**2**^	**inSeMP**^**2**^	**orgSeMP**^**2**^		
Initial body weight, kg	12.58	12.91	12.22	0.201	0.543
Final body weight, kg	23.7	24.7	23.4	0.759	0.455
ADG, kg/d	0.60	0.65	0.63	0.029	0.171
ADFI, kg/d	0.92	0.87	0.93	0.027	0.287
Gain:feed, kg/kg	0.67	0.75	0.69	0.026	0.058

*InSeMP* mushroom powder supplemented with inorganic selenium, *orgSeMP* mushroom powder enriched with organic selenium, *ADG* average daily gain, *ADFI* average daily feed intake

^1^Dietary treatments: (1) Basal diet; (2) basal diet + inSeMP containing an inorganic selenium (selenite) content of 0.3 mg/kg feed and a β-glucan content of 650 mg/kg feed; (3) basal diet + orgSeMP containing an organic selenium (selenocysteine) content of 0.3 mg/kg feed and a β-glucan content of 650 mg/kg feed

^2^A total of 8 replicates were used per treatment group

### Volatile fatty acids

The effects of dietary supplementation on the total concentrations of caecal VFA are presented in Table 5. Supplementation with inSeMP and orgSeMP decreased the concentration of BCVFA and the concentration of isovalerate compared to the basal group (*P* < 0.05).

**Table 5:** The effects of dietary treatment on total concentrations of VFA in the caecum (least-square mean values ± SEM)

VFA, mmol/L digesta	**Dietary treatments**^**1**^			**SEM**	***P*-value**
	**Basal**^**2**^	**inSeMP**^**2**^	**orgSeMP**^**2**^		
VFA, mmol/L digesta					
Total	176.79	180.01	175.87	17.439	0.985
Acetate	126.24	129.21	123.51	13.835	0.959
Propionate	32.23	33.91	34.00	2.732	0.876
Isobutyrate	0.69	0.51	0.50	0.098	0.333
Butyate	14.62	14.36	16.03	2.140	0.839
Isovalerate	1.27^b^	0.79^a^	0.64^a^	0.124	0.005
Valerate	1.75	1.23	1.19	0.192	0.098
Branched-chain VFA	3.71^b^	2.53^a^	2.33^a^	0.331	0.016

*InSeMP* mushroom powder supplemented with inorganic selenium, *orgSeMP* mushroom powder enriched with organic selenium, *VFA* volatile fatty acids

^1^Dietary treatments: (1) Basal diet; (2) basal diet + inSeMP containing an inorganic selenium (selenite) content of 0.3 mg/kg feed and a β-glucan content of 650 mg/kg feed; (3) basal diet + orgSeMP containing an organic selenium (selenocysteine) content of 0.3 mg/kg feed and a β-glucan content of 650 mg/kg feed

^2^A total of 8 replicates were used per treatment (experimental unit = pig)

^a−b^Mean values within a row with unlike superscript letters were significantly different (*P* < 0.05)

### Effects of mushroom powder supplementation on the caecal microbiota

#### Differential bacterial abundance analysis

All data on bacterial abundances at phylum, family and genus level are presented in Tables 6, 7 and 8. There were five bacterial phyla identified with Firmicutes being the dominant phyla (~ 79.6%) followed by Bacteriodetes (~ 14.64%), Proteobacteria (~ 1.34%) and Actinobacteria (~ 1.05%). The inSeMP and orgSeMP increased the relative abundance of Bacteriodetes compared to the basal group (*P* < 0.05).

**Table 6:** The effect of dietary treatment on the bacterial abundance (%) at phylum level (least-square mean values ± SEM)

Phylum	**Dietary treatments**^**1**^			**SEM**	***P*-value**
	**Basal**^**2**^	**inSeMP**^**2**^	**orgSeMP**^**2**^		
Bacteroidetes	8.87^a^	16.91^b^	18.14^b^	1.374	0.0002
Firmicutes	83.73	77.43	77.64	3.142	0.298
Actinobacteria	0.48	1.26	1.43	0.399	0.192
Proteobacteria	1.05	1.61	1.34	0.406	0.634

*InSeMP*, mushroom powder supplemented with inorganic selenium; *orgSeMP*, mushroom powder enriched with organic selenium

^1^Dietary treatments: (1) Basal diet; (2) basal diet + inSeMP containing an inorganic selenium (selenite) content of 0.3 mg/kg feed and a β-glucan content of 650 mg/kg feed; (3) basal diet + orgSeMP containing an organic selenium (selenocysteine) content of 0.3 mg/kg feed and a β-glucan content of 650 mg/kg feed

^2^A total of 8 replicates were used per treatment

^a−b^Mean values within a row with unlike superscript letters were significantly different (*P* < 0.05)

**Table 7:** The effect of dietary treatment on the bacterial abundance (%) at family level (least-square mean values ± SEM)

Family	**Dietary treatments**^**1**^			**SEM**	***P*-value**
	**Basal**^**2**^	**inSeMP**^**2**^	**orgSeMP**^**2**^		
Prevotellaceae	8.89^a^	17.83^b^	18.71^b^	1.3587	< .0001
Selenomonadaceae	0.09	0.40	0.44	0.188	0.467
Clostridiaceae	10.71	9.23	8.46	1.092	0.352
Lachnospiraceae	18.32	18.89	22.90	1.584	0.108
Hungateiclostridiaceae	1.06	0.66	0.64	0.334	0.584
Streptococcaceae	0.24	1.06	1.54	0.315	0.079
Lactobacillaceae	1.23^a^	1.74^a^	3.29^b^	0.502	0.031
Veillonellaceae	0.26	1.07	1.08	0.264	0.180
Ruminococcaceae	44.78^b^	37.50^ab^	34.81^a^	2.165	0.013
Acidaminococcaceae	0.98	1.06	0.78	0.349	0.839
Muribaculaceae	0.48	0.25	0.54	0.232	0.668
Erysipelotrichaceae	1.20	0.86	0.62	0.325	0.490
Oscillospiraceae	0.73	0.53	0.27	0.259	0.473

*InSeMP* mushroom powder supplemented with inorganic selenium, *orgSeMP* mushroom powder enriched with organic selenium

^1^Dietary treatments: (1) Basal diet; (2) basal diet + inSeMP containing an inorganic selenium (selenite) content of 0.3 mg/kg feed and a β-glucan content of 650 mg/kg feed; (3) basal diet + orgSeMP containing an organic selenium (selenocysteine) content of 0.3 mg/kg feed and a β-glucan content of 650 mg/kg feed

^2^A total of 8 replicates were used per treatment

^a−b^Mean values within a row with unlike superscript letters were significantly different (*P* < 0.05)

**Table 8:** The effect of dietary treatment on the bacterial abundance (%) at genus level (least-square mean values ± SEM)

Genus	**Dietary treatments**^**1**^			**SEM**	***P*-value**
	**Basal**^**2**^	**inSeMP**^**2**^	**orgSeMP**^**2**^		
*Blautia*	2.30	1.69	2.33	0.516	0.622
*Prevotella*	4.38^a^	10.77^b^	13.49^b^	1.111	< .0001
*Clostridium*	10.28	8.62	7.85	1.060	0.275
*Dorea*	1.24	2.56	1.82	0.471	0.191
*Coprococcus*	2.47	2.31	1.38	0.538	0.288
*Oscillibacter*	0.59	0.51	0.21	0.203	0.514
*Anaerobacterium*	0.90	0.66	0.61	0.321	0.772
*Streptococcus*	0.25	1.07	1.56	0.959	0.075
*Lactobacillus*	1.25^a^	1.75^ab^	3.33^b^	0.505	0.029
*Fournierella*	1.24	1.84	2.14	0.426	0.162
*Anaerobium*	0.96	0.61	0.57	0.266	0.399
*Mediterraneibacter*	0.91	0.66	0.63	0.285	0.734
*Eubacterium*	2.56	2.17	1.44	0.534	0.198
*Lachnobacterium*	0.48	0.31	0.41	0.248	0.534
*Dialister*	0.26	0.79	0.63	0.218	0.240
*Butyricicoccus*	0.38	0.58	0.65	0.265	0.865
*Gemmiger*	9.4	10.67	8.22	1.034	0.302
*Agathobacter*	4.82^a^	6.93^ab^	9.05^b^	0.871	0.016
*Faecalibacterium*	15.77	16.03	17.75	1.416	0.580
*Phascolarctobacterium*	0.97	0.97	0.78	0.343	0.942
*Kineothrix*	1.09	0.28	0.41	0.323	0.129
*Roseburia*	2.81^ab^	1.81^a^	4.40^b^	0.605	0.027
*Prevotellamassilia*	2.33	4.16	4.05	0.607	0.122
*Sporobacter*	10.31^b^	4.96^a^	3.87^a^	0.949	0.0001
*Ruminococcus*	5.88^b^	2.03^a^	2.50^a^	0.668	0.001
*Intestinimonas*	0.39	0.31	0.14	0.224	0.146
*Alloprevotella*	2.22	3.02	1.36	0.568	0.115

*InSeMP* mushroom powder supplemented with inorganic selenium, *orgSeMP* mushroom powder enriched with organic selenium

^1^Dietary treatments: (1) Basal diet; (2) basal diet + inSeMP containing an inorganic selenium (selenite) content of 0.3 mg/kg feed and a β-glucan content of 650 mg/kg feed; (3) basal diet + orgSeMP containing an organic selenium (selenocysteine) content of 0.3 mg/kg feed and a β-glucan content of 650 mg/kg feed

^2^A total of 8 replicates were used per treatment

^a−b^Mean values within a row with unlike superscript letters were significantly different (*P* < 0.05)

At family level, dietary supplementation with orgSeMP increased the relative abundance of Lactobacillaceae and decreased the relative abundance of Ruminococcaceae within the Firmicutes phylum compared to the basal group (*P* < 0.05). Dietary supplementation with orgSeMP and inSeMP increased the relative abundance of Prevotellaceae within the Bacteroidetes phylum (*P* < 0.05).

At the genus level, dietary supplementation with inSeMP and orgSeMP increased the relative abundance of *Prevotella* within the family Prevotellaceae and decreased the relative abundance of *Sporobacter* and *Ruminococcus* within the family Ruminococcaceae compared to the basal group (*P* < 0.05). Dietary supplementation with orgSeMP, increased the relative abundance of *Lactobacillus* within the family Lactobacillaceae, and *Agathobacter* within the family Lachnospiraceae compared to the basal group (*P* < 0.05). Supplementation with orgSeMP increased the relative abundance of *Roseburia* within the family Lachnospiraceae compared to the inSeMP group (*P* < 0.05).

### Gene expression in the small intestine

Differentially expressed genes are presented in Table 9. The complete gene expression data is presented in the supplementary materials (Tables S1).

**Table 9:** The effects of dietary treatment on the expression of nutrient transporters, immune markers and tight junctions in pigs duodenum, jejunum and ileum (least-square mean values ± SEM)

Item	**Gene**	**Dietary treatments**^**1**^			**SEM**	***P*-value**
		**Basal**^**2**^	**inSeMP**^**2**^	**orgSeMP**^**2**^		
Duodenum	*IL10*	1.04^a^	1.61^b^	1.03^a^	0.179	0.047
	*IL17a*	1.09	1.73	0.94	0.223	0.069
	*TLR4*	1.15^ab^	1.90^b^	0.87^a^	0.234	0.026
Jejunum	*MUC2*	1.34^ab^	1.49^b^	0.70^a^	0.211	0.038
	*SLC15A1*	1.13	0.73	1.13	0.153	0.099
	*FABP2*	1.22^b^	0.67^a^	1.13^b^	0.149	0.037
	*SLC2A2*	1.2	0.72	1.07	0.15	0.095
Ileum	*CLDN3*	0.84^ab^	1.38^b^	0.64^a^	0.347	0.008
	*MUC2*	0.72	0.73	0.91	0.189	0.089
	*DIO1*	0.35^a^	1.24^b^	0.79^ab^	0.169	0.011

*InSeMP* mushroom powder supplemented with inorganic selenium, *orgSeMP* mushroom powder enriched with organic selenium, *SLC15A1/PEPT1* peptide transporter 1, *FABP2*, fatty acid binding protein 2, *SLC2A2/GLUT2* glucose transporter 2, *IL10* interleukin 10, *MUC2* mucin 2, *IL17* interleukin 17, *TLR4* toll like receptor 4, *CLDN3* claudin 3, *DIO1* deiodinase type 1

^1^Dietary treatments: (1) Basal diet; (2) basal diet + unenriched mushroom powder (inSeMP) containing an inorganic selenium (selenite) content of 0.3 mg/kg feed and a β-glucan content of 650 mg/kg feed; (3) basal diet + selenium enriched mushroom powder (orgSeMP) containing an organic selenium (selenocysteine) content of 0.3 mg/kg feed and a β-glucan content of 650 mg/kg feed

^2^A total of 8 replicates were used per treatment

^a−b^Mean values within a row with unlike superscript letters were significantly different (*P* < 0.05)

In the duodenum, orgSeMP supplementation decreased the expression of *TLR4* compared to the inSeMP group (*P* < 0.05). The inSeMP supplementation increased the expression of *IL10* compared to all other groups (*P* < 0.05).

In the jejunum, inSeMP supplementation decreased the expression of *FABP2* compared to all other groups (*P* < 0.05). The orgSeMP supplementation decreased the expression of *MUC2* compared to the inSeMP group (*P* < 0.05).

In the ileum, orgSeMP reduced the expression of *CLDN3* compared to the inSeMP group (*P* < 0.05). The inSeMP supplementation increased the expression of *DIO1* compared to the basal group (*P* < 0.05).

### Small intestinal morphology

The effect of dietary supplementation on small intestinal morphology is presented in Table 10. In the duodenum, there was no difference in VH, CD and VH:CD among groups (*P* > 0.05). In the jejunum, pigs supplemented with inSeMP had decreased VH and VH:CD compared to the orgSeMP and basal group (*P* < 0.05). In the ileum, pigs supplemented with inSeMP had decreased VH compared to the orgSeMP and basal group (*P* < 0.05).

**Table 10:** Effect of dietary treatment on villus height and crypt depth in the small intestine (least-square mean values ± SEM)

**Item**	**Dietary treatments**^**1**^			**SEM**	***P*-value**
	**Basal**^**2**^	**inSeMP**^**2**^	**orgSeMP**^**2**^		
Duodenum					
VH, µm	306.55	283.11	304.50	12.648	0.366
CD, µm	136.16	133.48	140.18	7.611	0.823
VH:CD	2.31	2.17	2.19	0.142	0.778
Jejunum					
VH, µm	292.49^b^	227.79^a^	297.99^b^	15.567	0.007
CD, µm	138.30	131.89	143.19	8.408	0.641
VH:CD	2.15^b^	1.74^a^	2.10^b^	0.107	0.029
Ileum					
VH, µm	267.90^b^	216.84^a^	265.27^b^	15.600	0.053
CD, µm	134.41	118.23	127.05	8.832	0.445
VH:CD	2.03	1.89	2.12	0.129	0.457

*InSeMP* mushroom powder supplemented with inorganic selenium, *orgSeMP* mushroom powder enriched with organic selenium, *VH* villus height, *CD* crypt depth, *VH:CD* villus height to crypt depth ratio

^1^Dietary treatments: (1) Basal diet; (2) basal diet + inSeMP containing an inorganic selenium (selenite) content of 0.3 mg/kg feed and a β-glucan content of 650 mg/kg feed; (3) basal diet + orgSeMP containing an organic selenium (selenocysteine) content of 0.3 mg/kg feed and a β-glucan content of 650 mg/kg feed

^2^A total of 8 replicates were used per treatment group

^a−b^Mean values within a row with unlike superscript letters were significantly different (*P* < 0.05)

## Discussion

In the present study, it was hypothesised that orgSeMP would be more effective than inSeMP at enhancing gastrointestinal health parameters and modulating the caecal microbiota of post-weaned pigs, as organic sources of selenium exhibit lower toxicity and higher bioavailability in animals compared to inorganic sources [14]. In Period 1, dietary supplementation with orgSeMP improved FS compared to the basal group. In Period 2, dietary supplementation with orgSeMP increased the caecal abundance of bacterial members of the Firmicutes and Bacteroidetes phylum, including *Lactobacillus, Agathobacter, Roseburia* and *Prevotella* and decreased the abundance of *Sporobacter.* Dietary supplementation with inSeMP increased the caecal abundance of *Prevotella* and decreased the caecal abundance of *Sporobacter*. Dietary supplementation with orgSeMP and inSeMP reduced BCVFA in Period 2*.* The findings from this study indicate that orgSeMP supplementation was superior to inSeMP in supporting intestinal health through improving FS and promoting the development of a healthier microbiome composition in the caecum.

The post-weaning period in commercial pig production systems is characterised by reduced feed intake, growth rates and an increased incidence of diarrhoea [46]. In Period 1, dietary supplementation with orgSeMP reduced feed intake in pigs. A reduction in feed intake is only considered a negative outcome if it has an adverse effect on ADG, however ADG was unaffected in this study. In Period 2, dietary supplementation had no effect on growth performance. The lack of effects seen in the present study may be due to the good hygiene and husbandry practices which are observed in research facilities compared with commercial farms [47]. Previously, Rattigan et al. [48] observed differing responses of pigs to laminarin, a β-(1,3)-glucan from the seaweed *Laminaria* *digitata*, depending on the sanitary conditions suggesting that laminarin may have greater bioactivity under more challenging hygiene conditions. Thus perhaps, under more challenging conditions, a greater growth performance response would be observed in this study.

Dietary intervention with natural bioactives may play a role in shaping the structure and function of intestinal microbial communities. β-glucans are non-digestible polysaccharides and thus may enter the large intestine as a fermentation substrate for beneficial microorganisms [23]. Previous studies indicate that β-glucans can selectively stimulate the growth of beneficial bacteria and help to maintain the intestinal health [49, 50]. Selenium is an essential trace element that can modulate the gut microbiome and influence pig health [11]; however, its bioavailability is influenced by the chemical form of the selenium absorbed [51]. In the current study, supplementation with orgSeMP was superior to inSeMP in terms of modulating the caecal microbiota. This may be attributed to the source of selenium, as organic selenium is less toxic and more bioavailable for the animal [52]. Supplementation with orgSeMP altered the gut microbiota of the pigs by increasing the population of potentially beneficial bacteria, within the phylum Firmicutes, including *Lactobacillus and Agathobacter* compared to the basal group and *Roseburia* compared to the inSeMP group. Bacterial members of the genus *Lactobacillus* can enhance host gastrointestinal health through the competitive exclusion of pathogenic bacteria, producing antimicrobial peptides and enhancing immune function [53, 54]. In broilers, dietary supplementation with bacterial organic selenium was associated with increased caecal *Lactobacilli* spp. counts when compared to diets with inorganic selenium [10]. This further supports the advantageous effects of organic selenium over inorganic selenium on the gut microbiota. *Roseburia* and *Agathobacter* are beneficial gut bacteria that produce SCFAs, particularly butyrate [55, 56]. Thus, it may be anticipated that butyrate levels would be increased in the orgSeMP group, however caecal butyrate levels were unaffected in this study and unfortunately colonic butyrate levels were not measured.

InSeMP and orgSeMP supplementation increased the abundance of the phylum Bacteroidetes*,* including members *Prevotella* and decreased the abundance of the genera *Ruminococcus* and *Sporobacter,* within the phylum Firmicutes.* Sporobacter* is increased in pigs challenged with F4 ^+^ ETEC and may have a detrimental impact on gut health [57]. Both *Prevotella* and *Ruminococcus* are involved in the degradation of complex plant carbohydrates, thus their abundance usually increases in plant rich diets [58]. The reduced abundance of *Ruminococcus* in relation to *Prevotella* is an interesting finding. This may reflect differences between *Ruminococcus* and *Prevotella* in their utilization of carbohydrate substrates and that *Prevotella* in these pigs were primed towards mushroom β-glucan degradation. The increase in *Prevotella* in response to β-glucan supplementation is in agreement with previous studies investigating the microbial effects of yeast cell wall [59] and cereal β-glucans [60, 61].

FS remained within a healthy range throughout the duration of this study, which is likely attributed to the high health status of the animals used in this experiment. Nevertheless, orgSeMP improved FS compared to the basal group, and comparable to ZnO. These healthier FS were concurrent with a decrease in the concentration of caecal BCVFA. The inSeMP supplementation also reduced caecal BCVFA concentrations compared to the basal group but did not have the same effects as orgSeMP on FS. The improvement in FS in pigs supplemented with orgSeMP is likely attributed to the beneficial effects of organic selenium on the caecal microbiota, particularly the increased abundance of *Lactobacillus*. *Lactobacillus* can improve the immune response, intestinal function, and modulate the microbiome, which consequently, may improve faecal scores [62]. BCVFA are toxic metabolites associated with PWD and poor growth performance in pigs [63]. The reduced BCVFA in both mushroom groups is probably due to the β-glucan content of the mushrooms. In previous studies, pigs fed high fibre diets had reduced caecal [64] and colonic [65] BCVFA concentrations. β-glucans present in the mushrooms contributed to modulation of the caecal microbiota and it is likely that these microbial changes enhanced the utilization of amino acids as energy sources, thereby reducing the amount of BCVFA being produced. The improved FS in association with lower concentrations of BCVFA in the caecum, suggest that these orgSeMP-supplemented pigs had a healthier digestive tract compared to the basal group.

Gastrointestinal homeostasis is of utmost importance to the health of the weaned pig and disruption to this gives rise to intestinal inflammation. In the present study, inSeMP supplementation increased the gene expression of *TLR4* and had a tendency to increase the gene expression of *IL17* compared to the orgSeMP group. TLR4 is a pathogen recognition receptor and activation of TLR4 can lead to the production of proinflammatory cytokines, including IL-17, which has implications in the pathogenesis of chronic disease [66]. It is well known that increased expression of inflammatory cytokines compromise epithelial barrier function [67]. In the current study, the reduced villus height in the jejunum and ileum of inSeMP supplemented pigs, alongside the increased expression of *CLDN3* compared to the orgSeMP group, may be indicative of disruption of epithelial barrier integrity. Furthermore, inSeMP supplemented pigs had increased gene expression of *IL10*. IL-10 is an anti-inflammatory cytokine which is elevated in patients at early stages of infection, preceding elevations in pro-inflammatory cytokines [68]. As β-glucans are immunostimulatory compounds and initiate the inflammation process, it is possible that feeding β-glucans at high inclusion levels (650 ppm) may over activate the immune response, as observed in inSeMP supplemented pigs. In a previous study, dietary supplementation with 50 mg/kg of yeast β-glucans resulted in a slight immune response in weaned pigs, whereas increasing the β-glucan inclusion level to 200 mg/kg significantly enhanced the immune response [69]. It is worthy to note that supplementation with orgSeMP had a tendency to decrease expression of proinflammatory cytokine gene *IL6*; indicating a potential immunomodulatory effect of orgSeMP. Supplementation with orgSeMP attenuated the inflammatory response, and this is likely due to the source of selenium. These findings suggest that orgSeMP supplementation may be more effective at maintaining immune homeostasis compared to inSeMP supplementation.

## Conclusion

Dietary supplementation with orgSeMP reduced caecal BCVFA concentrations and improved the microbial population of pigs, represented by a higher abundance of several bacterial members, including *Lactobacillus*, *Agathobacter*, *Roseburia* and *Prevotella*. Dietary supplementation with orgSeMP was more effective at maintaining immune homeostasis compared to inSeMP supplementation. In conclusion, orgSeMP is a novel and sustainable way to incorporate selenium and β-glucans into the diet of weaned pigs whilst improving FS and modulating the caecal microbiota.

## Supplementary Information

**Additional file 1: Table S1.** The effects of dietary treatment on the expression of nutrient transporters, immune markers and tight junctions in pigs duodenum, jejunum and ileum.

## Data Availability

All data generated and/or analysed during this study are available from the corresponding author upon reasonable request.

## Abbreviations

ADFI: Average daily feed intake
ADG: Average daily gain
β-glucan: Beta-glucan
BW: Body weight
CCK: Cholecystokinin
CD: Crypt depth
CLDN1: Claudin 1
CLDN3: Claudin 3
CP: Crude protein
CXCL8/IL8: Interleukin 8
DM: Dry matter
FABP2: Fatty acid binding protein 2
G:F: Gain to feed ratio
GCN: Gene copy number
Ct: Threshold cycle
GE: Gross energy
GLM: General linearized model
IFNG: Interferon gamma
IL10: Interleukin 10
IL17: Interleukin 17
IL6: Interleukin 6
inSeMP: Mushroom powder supplemented with inorganic selenium
MUC1: Mucin 1
MUC2: Mucin 2
NDF: Neutral detergent fibre
orgSeMP: Mushroom powder enriched with organic selenium
PW: Post-weaning
QPCR: Quantitative real-time PCR
Se: Selenium
SLC15A1/PEPT1: Peptide transporter 1
SLC2A2/GLUT2: Glucose transporter 2
SLC2A5/GLUT5: Glucose transporter 5
SLC5A1/SGLT1: Sodium glucose linked transporter 1
TGFB1: Transforming growth factor beta 1
TLR4: Toll like receptor 4
TNFα: Tumor necrosis factor alpha
VFA: Volatile fatty acid
VH: Villous height
ZnO: Zinc oxide
