# Supplementary material for: The effects of dietary supplementation with mushroom or selenium enriched mushroom powders on the growth performance and intestinal health of post-weaned pigs
Source: J Anim Sci Biotechnol. 2023 Jan 11;14:12. doi: 10.1186/s40104-022-00808-x (PMC9832780; doi:10.1186/s40104-022-00808-x)
Supplement: Supplementary file 1 — Additional file 1: Table S1. The effects of dietary treatment on the expression of nutrient transporters, immune markers and tight junctions in pigs duodenum, jejunum and ileum. [file 40104_2022_808_MOESM1_ESM.docx]

**Table S1.** The effects of dietary treatment on the expression of nutrient transporters, immune markers and tight junctions in pigs duodenum, jejunum and ileum (Least-square mean values ± SEM)

|  | **Dietary treatments^1^** | | | | | |  |
| --- | --- | --- | --- | --- | --- | --- | --- |
| **Item** | **Gene** | **Basal^2^** | **inSeMP^2^** | **orgSeMP^2^** | **SEM** | **P-values** | |
| **Duodenum** |  |  |  |  |  |  | |
| Appetite regulators | *CCK* | 1.13 | 1.15 | 1.05 | 0.221 | 0.946 | |
|  | *NPY* | 1.37 | 1.13 | 0.90 | 0.279 | 0.502 | |
|  | *PYY* | 0.97 | 0.89 | 1.27 | 0.189 | 0.331 | |
|  | *GLP1* | 0.67 | 0.93 | 0.65 | 0.176 | 0.483 | |
| Tight junctions and immune markers | *CLDN1* | 1.37 | 1.13 | 0.90 | 0.279 | 0.502 | |
|  | *CLND3* | 0.81 | 1.19 | 1.50 | 0.321 | 0.352 | |
|  | *IL10* | 1.04^a^ | 1.61^b^ | 1.03^a^ | 0.179 | 0.047 | |
|  | *IL17a* | 1.09 | 1.73 | 0.94 | 0.223 | 0.069 | |
|  | *IL6* | 1.33 | 1.28 | 0.88 | 0.146 | 0.154 | |
|  | *CXCL8* | 1.22 | 1.34 | 1.12 | 0.170 | 0.825 | |
|  | *INFG* | 0.93 | 1.30 | 1.13 | 0.226 | 0.682 | |
|  | *TLR4* | 1.15^ab^ | 1.90^b^ | 0.87^a^ | 0.234 | 0.026 | |
|  | *TNFa* | 1.04 | 1.12 | 1.07 | 0.083 | 0.439 | |
|  | *MUC1* | 0.95 | 1.15 | 1.46 | 0.338 | 0.562 | |
|  | *MUC2* | 1.13 | 1.15 | 1.05 | 0.221 | 0.946 | |
|  | *SLC15A1* | 0.81 | 1.19 | 1.50 | 0.321 | 0.352 | |
| Nutrient transporters | *FABP2* | 0.78 | 1.19 | 1.20 | 0.151 | 0.120 | |
|  | *SLC2A2* | 0.93 | 1.19 | 0.97 | 0.156 | 0.472 | |
|  | *SLC2A5* | 1.00 | 1.18 | 1.31 | 0.900 | 0.777 | |
| Selenoproteins | *DIO1* | 0.97 | 0.89 | 1.27 | 0.189 | 0.331 | |
|  | *SelenoP* | 1.22 | 1.41 | 0.87 | 0.240 | 0.229 | |
|  | *TXNRD1* | 0.92 | 1.1 | 1.25 | 0.176 | 0.600 | |
| **Jejunum** |  |  |  |  |  |  | |
| Appetite regulators | *NPY* | 0.84 | 1.54 | 1.41 | 0.348 | 0.362 | |
|  | *PYY* | 1.09 | 1.42 | 0.72 | 0.205 | 0.123 | |
|  | *GLP1* | 0.95 | 1.23 | 0.88 | 0.162 | 0.281 | |
| Tight junctions and immune markers |  |  |  |  |  |  | |
|  | *CNDN1* | 0.89 | 1.25 | 1.30 | 0.232 | 0.442 | |
|  | *CLND3* | 1.11 | 1.24 | 0.90 | 0.159 | 0.391 | |
|  | *IL10* | 1.10 | 1.38 | 1.10 | 0.174 | 0.227 | |
|  | *IL17a* | 0.84 | 1.10 | 0.79 | 0.136 | 0.160 | |
|  | *IL6* | 0.89 | 1.59 | 1.54 | 0.392 | 0.480 | |
|  | *CXCL8* | 1.01 | 1.08 | 0.90 | 0.147 | 0.843 | |
|  | *INFG* | 0.78 | 1.03 | 1.15 | 0.183 | 0.564 | |
|  | *TLR4* | 1.31 | 0.76 | 0.96 | 0.173 | 0.107 | |
|  | *TNFa* | 0.93 | 0.96 | 0.99 | 0.030 | 0.404 | |
|  | *MUC1* | 1.25 | 1.69 | 0.56 | 0.336 | 0.126 | |
|  | *MUC2* | 1.34^ab^ | 1.49^b^ | 0.70^a^ | 0.211 | 0.038 | |
| Nutrient transporters | *SLC15A1* | 1.13 | 0.73 | 1.13 | 0.153 | 0.099 | |
|  | *FABP2* | 1.22^b^ | 0.67^a^ | 1.13^b^ | 0.149 | 0.037 | |
|  | *SLC2A2* | 1.20 | 0.72 | 1.07 | 0.150 | 0.095 | |
|  | *SLC2A5* | 1.31 | 0.76 | 0.96 | 0.173 | 0.107 | |
| Selenoproteins | *DIO1* | 2.03 | 0.92 | 0.95 | 0.464 | 0.190 | |
|  | *SelenoP* | 0.77 | 1.08 | 1.87 | 0.187 | 0.329 | |
|  | *TXNRD1* | 0.78 | 0.82 | 1.15 | 0.150 | 0.174 | |
| **Ileum** |  |  |  |  |  |  | |
| Appetite regulators | *NPY* | 1.21 | 1.13 | 0.86 | 0.410 | 0.196 | |
|  | *PYY* | 0.93 | 1.61 | 0.87 | 0.09 | 0.221 | |
|  | *GLP1* | 1.21 | 1.91 | 1.32 | 0.247 | 0.154 | |
| Tight junctions and immune markers | *CLDN1* | 1.21 | 1.13 | 0.86 | 0.410 | 0.196 | |
|  | *CLDN3* | 0.84^ab^ | 1.38^b^ | 0.64^a^ | 0.347 | 0.008 | |
|  | *IL10* | 1.13 | 1.42 | 1.28 | 0.545 | 0.232 | |
|  | *IL17a* | 1.00 | 1.55 | 0.81 | 0.365 | 0.500 | |
|  | *IL6* | 0.77 | 1.34 | 1.36 | 0.290 | 0.265 | |
|  | *CXCL8* | 1.30 | 1.13 | 1.07 | 0.867 | 0.248 | |
|  | *INFG* | 1.03 | 1.16 | 1.06 | 0.908 | 0.130 | |
|  | *MUC1* | 0.99 | 0.94 | 0.99 | 0.040 | 0.672 | |
|  | *MUC2* | 0.72 | 0.73 | 0.91 | 0.189 | 0.089 | |
|  | *TLR4* | 1.30 | 1.53 | 1.46 | 0.954 | 0.297 | |
|  | *TNFa* | 0.88 | 1.14 | 1.16 | 0.618 | 0.204 | |
| Nutrient transporters | *SLC15A1* | 1.83 | 1.18 | 1.00 | 0.299 | 0.269 | |
|  | *FABP2* | 2.21 | 1.37 | 1.14 | 0.145 | 0.362 | |
|  | *SLC2A2* | 1.72 | 1.41 | 0.96 | 0.306 | 0.232 | |
|  | *SLC2A5* | 1.63 | 1.17 | 0.78 | 0.296 | 0.164 | |
| Selenoproteins | *DIO1* | 0.35^a^ | 1.24^b^ | 0.79^ab^ | 0.169 | 0.011 | |
|  | *SelenoP* | 1.19 | 1.61 | 1.12 | 0.213 | 0.350 | |

InSeMP, mushroom powder supplemented with inorganic selenium; orgSeMP, mushroom powder enriched with organic selenium; *SLC15A1/PEPT1*, peptide transporter 1; *FABP2*, fatty acid binding protein 2; *SLC2A1/GLUT1*, glucose transporter 1; *SLC5A1/SGLT1*, sodium glucose linked transporter 1; *SLC2A2/GLUT2*, glucose transporter 2; *SLC2A5/GLUT5*, glucose transporter 5; *CCK*, cholecystokinin; *TNF*, tumor necrosis factor alpha*; CXCL8*, interleukin 8; *IL6*, interleukin 6; *IL10*, interleukin 10; *IFNG*, interferon gamma; *ZO1*, zonulin; *MUC2*, mucin 2; TGF-β, transforming growth factor beta*; IL17*, interleukin 17; *TLR4*, toll like receptor 4; *CLDN3*, claudin 3; *CLDN1*, claudin 1; *MUC1*, mucin 1; DIO1, Deiodinase Type 1; SelenoP, Selenoprotein P; TXNRD1, thioredoxin reductase 1.

^1^Dietary treatments: (1) Basal diet; (2) basal diet + unenriched mushroom powder containing an inorganic selenium (selenite) content of 0.3 mg/kg feed and a β-glucan content of 650 mg/kg feed; (3) basal diet + selenium enriched mushroom powder containing an organic selenium (selenocysteine) content of 0.3 mg/kg feed and a β-glucan content of 650 mg/kg feed.

^a-b^Mean values within a row with unlike superscript letters were significantly different (p < 0.05).

^2^A total of 8 replicates were used per treatment.
